# Geometric and dosimetric evaluation of auto-segmentation of brain arteriovenous malformations using multimodal imaging in stereotactic radiosurgery

**DOI:** 10.3389/fnins.2025.1645990

**Published:** 2025-10-30

**Authors:** Xing Di, Wenqian Xu, Xiu Gong, Minghao Sun, Tao Jin, Yike Xu, Lei Zhu, Huaguang Zhu, Guanghai Mei, Xiaoxia Liu

**Affiliations:** ^1^Department of Neurosurgery, Huashan Hospital, Fudan University, Shanghai, China; ^2^Neurosurgical Institute of Fudan University, Shanghai, China; ^3^Department of Oncology, Qingdao Central Hospital, University of Health and Rehabilitation Sciences, Qingdao, Shandong, China; ^4^Department of Radiation Oncology Physics and Technology, Shandong Cancer Hospital and Institute, Shandong First Medical University and Shandong Academy of Medical Sciences, Jinan, Shandong, China

**Keywords:** brain arteriovenous malformations, white matter tracts, stereotactic radiosurgery, deep learning, auto-segmentation

## Abstract

**Background:**

Optimizing radiation dose to protect white matter (WM) tracts during stereotactic radiosurgery (SRS) of brain arteriovenous malformations (bAVMs) necessitates the integration of diffusion tensor imaging (DTI)-based WM tractography to delineate WM tracts and establish dose constraints. Conventional manual delineation of perilesional targets demonstrated significant operational inefficiency, primarily attributed to the complex structural interdigitation between pathological vasculature and eloquent brain areas.

**Purpose:**

This study aimed to develop a two-stage deep learning (DL) method that combines a two-dimensional (2D) U-Net detection-aided and three-dimensional (3D) self-attention segmentation model for automatic bAVM segmentation. This method focuses on improving efficiency in clinical practice while protecting WM tracts using multimodal imaging and WM tractography in SRS.

**Methods:**

We analyzed imaging data from 191 patients who underwent CyberKnife-based SRS at Huashan Hospital, Fudan University, with bAVMs closely adjacent to WM tracts. A total of 153 patients were used to construct a two-stage DL model to segment the bAVMs on multimodal imaging and WM tractography, while the remaining 38 patients were utilized to validate the model's performance. We introduced spatial and channel attention modules in the U-Net variant, along with a versatile “Attentional ResBlock,” achieving parameter efficiency through cross-dimensional interaction while preserving model fidelity. The accuracy of the auto-segmented contours is evaluated using geometric indices and dosimetric endpoints.

**Results:**

Our proposed model demonstrated superior segmentation performance, achieving a dice similarity coefficient (DSC) of 0.84 ± 0.05, sensitivity of 0.92 ± 0.09, and F2-score of 0.79 ± 0.08. Furthermore, it attained a low Hausdorff distance (4.55 ± 1.14 mm) and mean surface distance (0.53 ± 0.08 mm), indicating exceptional boundary delineation precision. The difference in the proportion of WM tracts within the target region between manual and our automated contours is minimal (0.08 ± 0.13). Meanwhile, strong concordance is observed between auto-segmented and manually contoured targets across the majority of dosimetric endpoints, with a mean difference of 0.46 Gy. The received dose of WM tracts in the two comparison plans also has an acceptable representation of dosimetric parameters (*R*^2^ = 0.92 for Dmean and 0.88 for V1Gy). Dose exposition of the organ at risk (OAR) shows no statistically significant differences in treatment plans with auto-segmentation targets compared to regular plans.

**Conclusion:**

The reliable bAVM automated-segmentation method has been validated and may support SRS planning for bAVMs and thus avoid neurological sequelae after SRS in considering WM tracts protection.

## 1 Introduction

Brain arteriovenous malformations (bAVMs) represent congenital cerebrovascular disorders pathologically defined by direct shunts between arterial and venous systems without intervening capillaries, resulting in hemodynamic compromise and hemorrhagic predisposition (Lawton et al., 2015; Rutledge et al., 2021; Rammos et al., 2016). Modern stereotactic radiosurgery (SRS) modalities enable less-fraction delivery of radiation doses (20–25 Gy) to bAVMs' nidal volumes (< 3 cm^3^), achieving 70–90% 3-year occlusion rates with < 5% symptomatic radiation necrosis incidence (Samaniego et al., 2024; Ahmed et al., 2025; Graffeo et al., 2020; Kano et al., 2012). The dosimetric imperative of delivering radiation doses necessitates a precise definition of therapeutic margins during bAVM SRS, requiring simultaneous optimization of target coverage and functional preservation in eloquent cortices mediating language, visuospatial, and sensorimotor processing (Buis et al., 2007; Ding et al., 2013; Hadjipanayis et al., 2001).

Accurate localization of eloquent regions and white matter (WM) tracts within the bAVM margin is therefore critical for preventing neurological sequelae (Maruyama et al., 2008; Ellis et al., 2012). Recently, diffusion tensor imaging (DTI)-based WM tractography has been integrated into SRS treatment planning (Pantelis et al., 2010; Gavin and Sabin, 2016; Altabella et al., 2018; Unnikrishnan et al., 2021; Yahya and Manan, 2019). This integration facilitates identification of the anatomical relationship between WM tracts and the nidus, thereby enabling optimization of treatment plan design. Precise identification and delineation of the bAVMs constitute essential steps in SRS planning. Currently, targets are manually identified by neuroradiologists and delineated by radiation oncologists using multimodal imaging combined with WM tractography. However, conventional workflows demonstrated resource-intensive operator-dependent characteristics compounded by inter-rater inconsistencies, particularly when managing targets juxtaposed to critical WM tracts.

Given that deep learning (DL) algorithms represent the forefront of medical image analysis (Li et al., 2020; Park et al., 2019; Liang et al., 2020; Almberg et al., 2022; Peng et al., 2019), auto-segmentation techniques have been developed to overcome the limitations of manual bAVM contouring by providing efficient and observer-independent results. Jiao et al. proposed a U-Net model to detect and quantify nidus diffuseness in bAVMs using magnetic resonance angiography (MRA) images (Jiao et al., 2022). Similarly, Hong et al. implemented YOLOv5 and YOLOv8 algorithms to detect bAVMs and incorporated pre-training techniques to improve bAVMs' segmentation performance with limited datasets (Hong et al., 2024). While Wang et al. developed an intensely supervised V-Net for delineating the bAVMs from CT images and evaluated the resultant target dose coverage changes (Wang et al., 2019). These studies collectively validate the reliability of DL for direct segmentation of bAVMs in medical imaging.

However, significant limitations persist. Existing methodologies primarily rely on single-modal imaging, which may inadequately capture the complex morphology and characteristics of bAVMs. Furthermore, lesions proximal to WM tracts often receive excessive radiation doses, largely due to challenges in their precise identification on CT or conventional MRI. Finally, while geometric indices serve as valuable metrics for assessing segmentation accuracy, the dosimetric data derived from auto-segmented contours represent a significantly more meaningful endpoint. Therefore, a systematic dosimetric analysis of auto-segmented bAVMs and critical structures, such as WM tracts, also needs consideration.

Given the limitations mentioned above, we have developed an innovative two-stage DL ensemble, which integrates a two-dimensional (2D) U-Net detection-aided and a three-dimensional (3D) self-attention network for automatic bAVM segmentation. This approach leverages multimodal imaging alongside WM tractography, marking the first utilization of WM tractography in the automated segmentation of bAVMs. It aims to improve the precision of bAVM data extraction and ensure the preservation of WM tracts. The results of bAVM auto-segmentations are evaluated for standard geometric indices, including dice similarity coefficient (DSC), Sensitivity, F2-score, Hausdorff distance (HD), and mean surface distance (MSD). The various dosimetric parameters in the target are obtained to assess the dosimetric differences of the proposed method. Finally, we conduct a comparative analysis of dosimetric outcomes between two contrast treatment plans based on auto-segmented contours and manual contours. This comparison focuses on the accuracy of dosimetric endpoints to validate the performance and reliability of our proposed auto-segmentation method.

## 2 Methods and materials

### 2.1 Clinical dataset

A retrospective analysis was conducted on 191 patients with bAVMs located adjacent to WM tracts, all of whom underwent CyberKnife-based SRS in Huashan Hospital, Fudan University, from 2016 to 2023. This study strictly complies with the ethical principles outlined in the Declaration of Helsinki and received approval from the local institutional review board of Huashan Hospital, Fudan University. Demographic and clinical characteristics, including age, sex, lesion size, affected hemisphere, and Spetzler–Martin (SM) grading (Spetzler and Martin, 2008) are documented in Table 1. All manual contours are rigorously reviewed and verified by experienced neuroradiologists to ensure quality control. Patients are immobilized with customized thermoplastic masks and undergo computed tomography (CT) simulation using a Toshiba 64-slice scanner (Japan). Axial images are acquired from the vertex to the chin at a slice thickness of 1 mm. The 3D time-of-flight magnetic resonance angiography (TOF-MRA) and T2-weighted magnetic resonance imaging (MRI) images are acquired using a 3.0T MAGNETOM Trio scanner (Siemens, Germany). The TOF-MRA sequence is performed with a repetition time (TR) of 22 ms, echo time (TE) of 3.5 ms, flip angle of 18°, and slice thickness of 1 mm. The T2-weighted images are obtained using a turbo spin–echo fluid-attenuated inversion recovery (FLAIR) sequence with a repetition time of 9,000 ms, echo time of 90 ms, inversion time of 2,500 ms, flip angle of 150°, and slice thickness of 1 mm. WM tractography and T1-weighted MRI are performed in the intraoperative MRI suite using a MAGNETOM Verio scanner (Siemens AG, Germany). Image acquisition and analysis are conducted using the Neuro 3D Analysis workstation. Anatomical imaging employed a 3D magnetization-prepared rapid acquisition gradient echo (MPRAGE) sequence, acquiring whole-brain axial T1-weighted images with the following parameters: slice thickness 1 mm; TR, 1,900 ms; TE, 2.98 ms; flip angle 90°; and isotropic voxel size 1.0 mm × 1.0 mm × 1.0 mm. The field of view was 256 mm with a 256 × 256 matrix. Diffusion tensor imaging utilized a single-shot, multi-slice 2D spin–echo planar imaging sequence with diffusion sensitization and fat suppression. DTI parameters included: axial acquisition with 42 slices, 2 mm slice thickness, no gap, TR 9,900 ms, TE 90 ms, voxel size 1.5 mm × 1.5 mm × 3 mm3, flip angle 90°, and field of view 240 mm with a 128 × 128 matrix, providing full brain coverage. The 191 patients are divided into training and testing groups with a ratio of approximately 80:20 (153:38). Image registration of each patient's MRI and DTI volumes to their corresponding CT image is accomplished using a proprietary MIM workflow (Fukumitsu et al., 2017). To enhance the robustness of the model, data augmentation such as rotation, flipping, and zooming is employed, facilitating the model's ability to learn transformation-invariant features. Finally, the preprocessed CT and MRI data are converted to NumPy arrays and loaded into PyTorch, ready for model training (Van Der Walt et al., 2011; Paszke, 2019).

**Table 1:** Demographic and clinical characteristics of bAVM patients.

**Dataset Type**	**Age, median [IQR]**	**Male sex**	**Size (cc), median [IQR]**	**SM grading, number (%)**		
				**II**	**III**	**IV**
Training set (*n =* 153)	26 [17–62]	87 (56.9%)	15.4 [1.3–55.9]	61 (39.9%)	84 (54.9%)	8 (5.2%)
Test Set (*n =* 38)	25 [19–48]	20 (52.6%)	15.1 [1.5–48.6]	14 (36.8%)	23 (60.5%)	1 (2.6%)

SM, Spetzler–Martin grading.

### 2.2 Segmentation

#### 2.2.1 Training process

**Figure 1:**
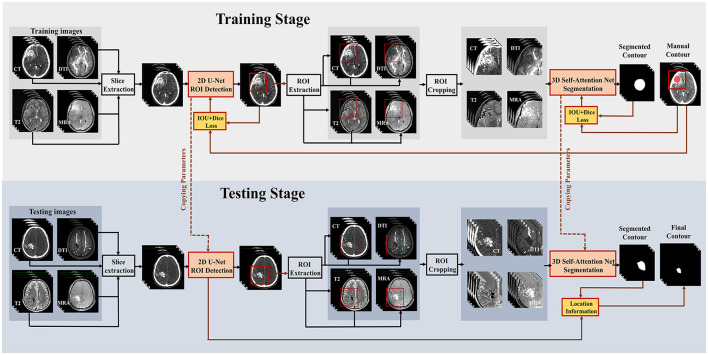
The schematic flow diagram of the proposed method. The **upper part** illustrates the training stage, while the **lower part** shows the testing stage for new data.

A two-stage deep learning ensemble is trained to segment bAVM nidus, which consists of a 2D U-Net detection model to localize the bAVM regions of interest (ROI) and a 3D self-attention network to segment bAVMs within the identified location. The whole process for the training and testing phase is illustrated in Figure 1. In the first stage of the training phase, the multimodal images of each training patient are served as input images and concatenated as multi-channel inputs, with the manual contours serving as the target for learning. The binary mask of bAVMs is coarsely generated in a 2D U-Net, and then a 128 × 128 × 128 pixel ROI bounding box centered at the mass center of the mask is created to localize the bAVM nidus. The coordinates of these cropped ROIs are stored and subsequently utilized to map the predicted masks back to the original images. In the second stage, the multimodal images are cropped to the defined ROI, and these images are then input into the 3D self-attention architecture, which provides accurate and efficient bAVM segmentation within the ROI. The 2D U-Net and 3D self-attention network are implemented using Python 3.8 with PyTorch 1.12, enhanced by CUDA 12.2, on six NVIDIA RTX 4080 Ti GPUs. The models undergo training and testing across 300 epochs, employing the Adam optimizer, which incorporates a momentum term of 0.5 to enhance convergence. The initial learning rate is set at 0.0002 and is systematically reduced by half whenever the error rates on the validation set plateau, ensuring efficient and effective learning progression.

#### 2.2.2 Network architecture

**Figure 2:**
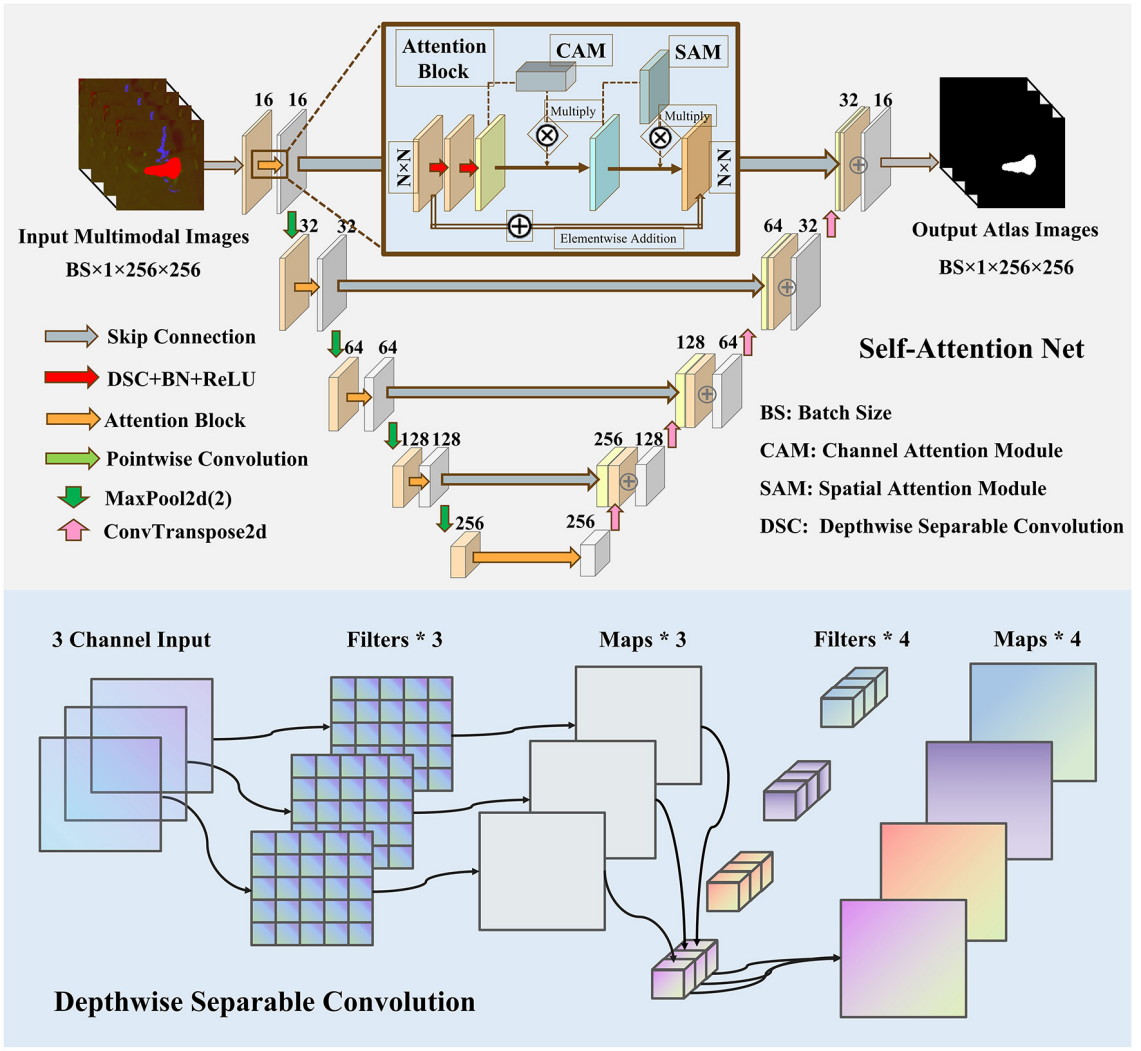
The proposed architecture of the self-attention network **(top)** and the schematic diagram of depthwise separable convolution **(bottom)**.

The architecture of our proposed 3D self-attention network is detailed in Figure 2, top, and it is divided into two stages: encoding and decoding. The encoding stage consists of five levels arranged in a hierarchical structure. The input image slices, which were previously cropped during the detection phase, undergo processing via combined convolution and attention blocks. Max-pooling is then applied to down-sample the feature maps. To maintain the multi-level features effectively, concatenation is employed to enhance the volume of feature maps from both the encoding and decoding stages. The final transformation involves funneling these maps through a convolution layer to decrease their dimensionality to two channels. This is immediately followed by a “tanh” activation layer that serves to polarize the feature maps, thereby distinguishing the posterior probabilities of the bAVMs from those of normal tissue.

#### 2.2.3 Attentional block

Inspired by CBAM (Ye et al., 2018), the attention mechanism is embedded within the U-Net architecture to improve edge prediction accuracy for bAVM segmentation. The proposed attention mechanism comprises two specialized submodules: a channel attention module (CAM) for feature refinement and a spatial attention module (SAM) for contextual weighting. The CAM utilizes dual-pooling operations (global average pooling and global max pooling) to integrate multi-scale feature representations. These aggregated features transform a shared multilayer perceptron (MLP). Subsequent element-wise summation and sigmoid activation yield channel-wise weighting coefficients (*V*_c_), which rescale the original feature map (*F*) via Hadamard product. The SAM then prioritizes discriminative spatial regions by generating attention weights through a lightweight convolutional subnetwork applied to the refined features, followed by sigmoid normalization. This hierarchical attention framework progressively refines feature discriminability for boundary-critical structures. The new feature can be obtained by multiplying the weight coefficient with the original feature graph *F*. To optimize computational efficiency and model compactness, we implement depth-wise separable convolution as a parameter-reduction strategy, as schematically depicted by the red arrow in Figure 2, attention block. This architectural innovation decomposes standard convolution into two sequential operations: depth-wise separable convolution that performs spatial filtering through channel-wise independent kernels (Chollet, 2017), followed by point-wise convolution (1 × 1 kernels) for channel combination. The decoupled processing mechanism achieves substantial parameter reduction compared with conventional convolution. The schematic diagram of the depth-wise separable convolution is illustrated in Figure 2, bottom. The full stages of CAM and SAM are shown as follows:


(1) $$\begin{array}{l} V_{c}\left(F\right)=\sigma \left(MLP\left(AvgPool\left(F\right)\right)+MLP\left(MaxPool\left(F\right)\right)\right)\\ \;=\sigma (W_{1}\left(W_{0}\left(F_{avg}^{c}\right)\right)+\;\left(W_{1}\left(W_{0}\left(F_{\max}^{c}\right)\right)\right) \end{array}$$

(2) $$\begin{array}{l} V_{s}\left(F\right)=\sigma \left(f\left(\left[AvgPool\left(F\right),\;MaxPool\left(F\right)\right]\right)\right)\\ \;=\sigma \left(f\left(\left[F_{avg}^{c}\;;\;F_{\max}^{c}\right]\right)\right) \end{array}$$

where *MLP* represents the multilayer perceptron, *F* represents the original feature, *V*_*c*_ and *V*_*s*_ represent the weight coefficient for CAM and SAM, respectively.

#### 2.2.4 Loss functions

Recent studies have predominantly employed either the Intersection over Union (IoU) or Dice loss functions within their neural network architectures (Jadon, 2020; Sudre et al., 2017). In this work, we introduce a novel compound loss function designed to supervise the training of our network. This function integrates both IoU and Dice losses, strategically penalizing discrepancies while simultaneously promoting congruence between the predicted segmentations and the ground truth data. The loss formulations for the IoU and Dice metric are as follows:


(3) $$\begin{array}{l} L_{IoU}=1-IoU=1-\frac{TP}{TP+FP+FN} \end{array}$$

(4) $$\begin{array}{l} L_{Dice}=1-\frac{2\times TP}{2\times TP+FP+FN} \end{array}$$

where TP, FP, and FN are true positive, false positive, and false negative, respectively.

By combining these two distinct loss functions, we establish the composite loss function for deep supervision as follows:


(5) $$\begin{array}{l} L_{final}=\frac{L_{IoU}+L_{Dice}}{2} \end{array}$$

### 2.3 Evaluation metrics

Quantitative comparisons between the segmentation results from our proposed model and manual contouring are conducted for each patient in the validation dataset, employing metrics such as DSC, sensitivity, and F2-score (Dice, 1945; Altman et al., 1994; Devarriya et al., 2020). Several established architectures, including U-Net (Montazerolghaem et al., 2023) and Res-Net (Targ et al., 2016), along with the state-of-the-art nnU-Net (Isensee et al., 2021) framework, are evaluated to demonstrate the superior performance of our proposed network. To quantitatively evaluate the contribution of the attention modules, an ablation study is conducted. We compare the performance of the complete proposed model (with attention modules) against an ablated variant, where all attention modules are removed. The metrics employed in this study are defined as follows:


(6) $$\begin{array}{l} DSC\left(A,B\right)=\frac{2|A\cap B|}{\left|A|+|B\right|}=\frac{2TP}{2TP+FP+FN} \end{array}$$

(7) $$\begin{array}{l} Sensitivity=\frac{TP}{TP+FN} \end{array}$$

(8) $$\begin{array}{l} F2=\frac{5TP}{5TP+4FP+FN} \end{array}$$

(9) $$\begin{array}{l} HD\left(A,B\right)=\max\;\left(h\left(A,B\right),h\left(B,A\right)\right) \end{array}$$

(10) $$\begin{array}{l} MSD\left(A,B\right)=\frac{h\left(A,B\right)+h\left(B,A\right)}{2} \end{array}$$

where TP, FP, and FN are true positive, false positive, and false negative, respectively. $h\left(A,B\right)=\max_{a\in A}\min_{b\in B}||a-b||$, $h\left(B,A\right)=\max_{b\in B}\min_{a\in A}||b-a||$, ||.|| denotes the Euclidean distance. A and B represent auto-segment and manual contours, respectively.

The WM tracts in target (WMT) is used to evaluate the proportion of WM tracts delineated within the target region. It is defined as follows:


(11) $$WMT=\left|\frac{A\cap WM}{A}-\frac{B\cap WM}{B}\right|$$

where A and B represent auto-segment and manual contours, respectively. WM is the contours of white matter tracts.

The dose-volume histograms (DVH) and various dosimetric parameters are derived for each patient from both auto-segmented and manually contoured bAVMs' regions to assess the dosimetric implications of the proposed segmentation method. The dosimetric endpoints evaluation, includes the mean dose (Dmean), maximum dose (Dmax), 99% target volume dose (D99), and 95% target volume dose (D95). To evaluate the variation in dose permissible to the WM tracts, while ensuring adequate coverage and conformality of the bAVM target, two controlled treatment plans are designed for each patient in the test dataset using the CyberKnife TPS (version 4.6, Accuray, Inc., Sunnyvale, USA). These plans are designed by a senior physician, based on auto-segmented and manually contoured bAVMs' outlines. To maintain comparability of dosimetric endpoints, all other planning parameters such as prescription dose, fraction, and size of collimators are held constant (shown in Supplementary Table S1). The correlation between dosimetric endpoints derived from WM tracts in these two plans is assessed using the *R*^2^ value of the linear regression.

## 3 Results

The overall geometric parameters for all 38 patients in the test dataset and model evaluation are summarized in Table 2. Our proposed model achieved superior performance, with mean DSC, Sensitivity, and F2-score values of 0.84 ± 0.05, 0.92 ± 0.09, and 0.79 ± 0.08, respectively, outperforming all compared methods including nnU-Net (0.79 ± 0.13, 0.88 ± 0.13, 0.68 ± 0.17), U-Net (0.76 ± 0.10, 0.81 ± 0.16, 0.70 ± 0.11), and Res-Net (0.73 ± 0.14, 0.77 ± 0.20, 0.67 ± 0.14). Moreover, our method also attained the lowest HD (4.55 ± 0.56 mm) and MSD (0.53 ± 0.08 mm), indicating a notable improvement in segmentation boundary precision. The difference in the proportion of WM tracts within the target region between manual and our auto-segmented contours is minimal, with a recorded value of 0.08 ± 0.13. The corresponding values are 0.15 ± 0.24 for Res-Net, 0.12 ± 0.32 for U-Net, and 0.13 ± 0.20 for nnU-Net, respectively.

**Table 2:** Quantitative results for the segmentation of different methods.

**Segmentation Model**	**DSC**	**Sensitivity**	**F2-score**	**HD**	**MSD**	**WMT**
Res-Net	0.73 ± 0.14	0.77 ± 0.20	0.67 ± 0.14	5.34 ± 1.27	0.83 ± 0.13	0.15 ± 0.24
U-Net	0.76 ± 0.10	0.81 ± 0.16	0.70 ± 0.11	5.51 ± 1.48	0.78 ± 0.16	0.12 ± 0.32
nnU-Net	0.79 ± 0.13	0.88 ± 0.13	0.68 ± 0.17	5.07 ± 1.21	0.70 ± 0.11	0.13 ± 0.20
Ours	0.84 ± 0.05	0.92 ± 0.09	0.79 ± 0.08	4.55 ± 0.56	0.53 ± 0.08	0.08 ± 0.13

**Figure 3:**
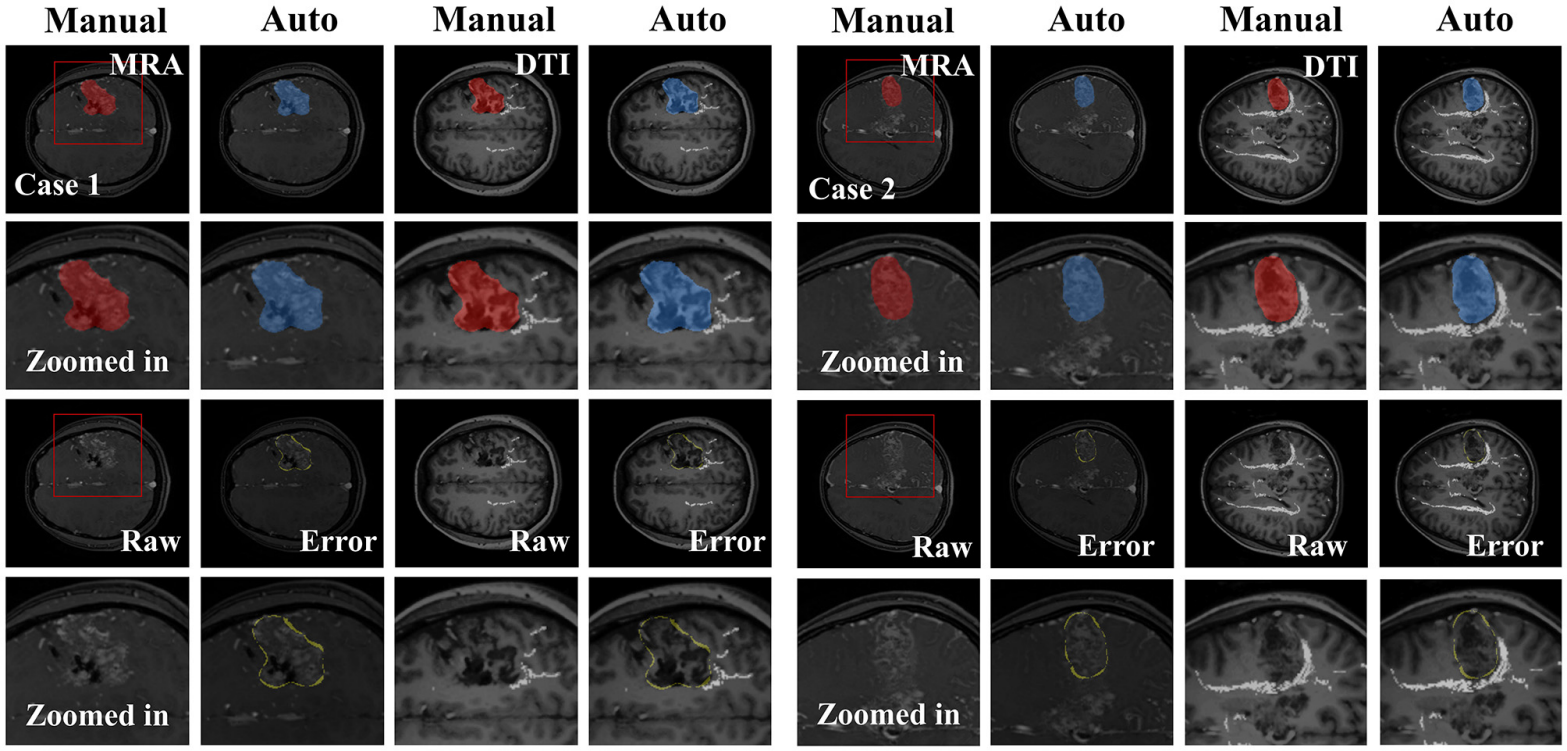
Segmentation results for two tested cases in DTI and MRA views. The middle row shows the zoomed-in images of full-view DTI images. The zoomed-in window location is indicated by a red rectangle.

The results of the ablation study on the attention mechanisms are summarized in Table 3. Removing the attention modules led to a noticeable degradation in segmentation performance across all metrics. Specifically, the model with attention modules achieved a superior DSC of 0.84 ± 0.05, compared to 0.80 ± 0.09 for the ablated version. Similarly, the inclusion of attention mechanisms reduced the HD from 5.13 ± 0.85 mm to 4.55 ± 0.56 mm and the MSD from 0.65 ± 0.14 mm to 0.53 ± 0.08 mm. A detailed breakdown of segmentation performance across Spetzler–Martin grades is provided in Table 4. The model achieved consistent and high DSC across all grades (Grade II: 0.839 ± 0.035; Grade III: 0.842 ± 0.031; Grade IV: 0.831), demonstrating its robustness in segmenting bAVMs of varying complexity. Similar trends are observed in sensitivity, F2-score, and other boundary distance metrics. These quantitative results validate the high accuracy of contours delineated by our proposed segmentation method. Two examples of manual and predicted contours are illustrated in Figure 3, where manual contours are depicted in red, auto-segmented contours in blue, and discrepancies are highlighted in yellow masks. From the zoomed-in images, it is evident that the target area delineated by the model effectively avoided WM tracts, closely matching the performance of manual delineation.

**Table 3:** Results of the ablation study on attention mechanisms.

**Model variant**	**DSC**	**HD**	**MSD**
With attention modules	0.84 ± 0.05	4.55 ± 0.56	0.53 ± 0.08
Without attention modules	0.80 ± 0.09	5.13 ± 0.85	0.65 ± 0.14

**Table 4:** Quantitative evaluation of segmentation performance across Spetzler–Martin grades.

**S-M grades**	**DSC**	**Sensitivity**	**F2-score**	**HD**	**MSD**
II	0.839 ± 0.035	0.939 ± 0.059	0.774 ± 0.081	4.394 ± 0.590	0.527 ± 0.074
III	0.842 ± 0.031	0.908 ± 0.100	0.791 ± 0.079	4.599 ± 0.492	0.539 ± 0.062
IV	0.831	0.981	0.847	4.751	0.541

Grade IV results are presented as mean values due to the limited sample size. Data in this table are presented with three decimal places to better illustrate the differences between groups.

SM, Spetzler–Martin grading.

**Figure 4:**
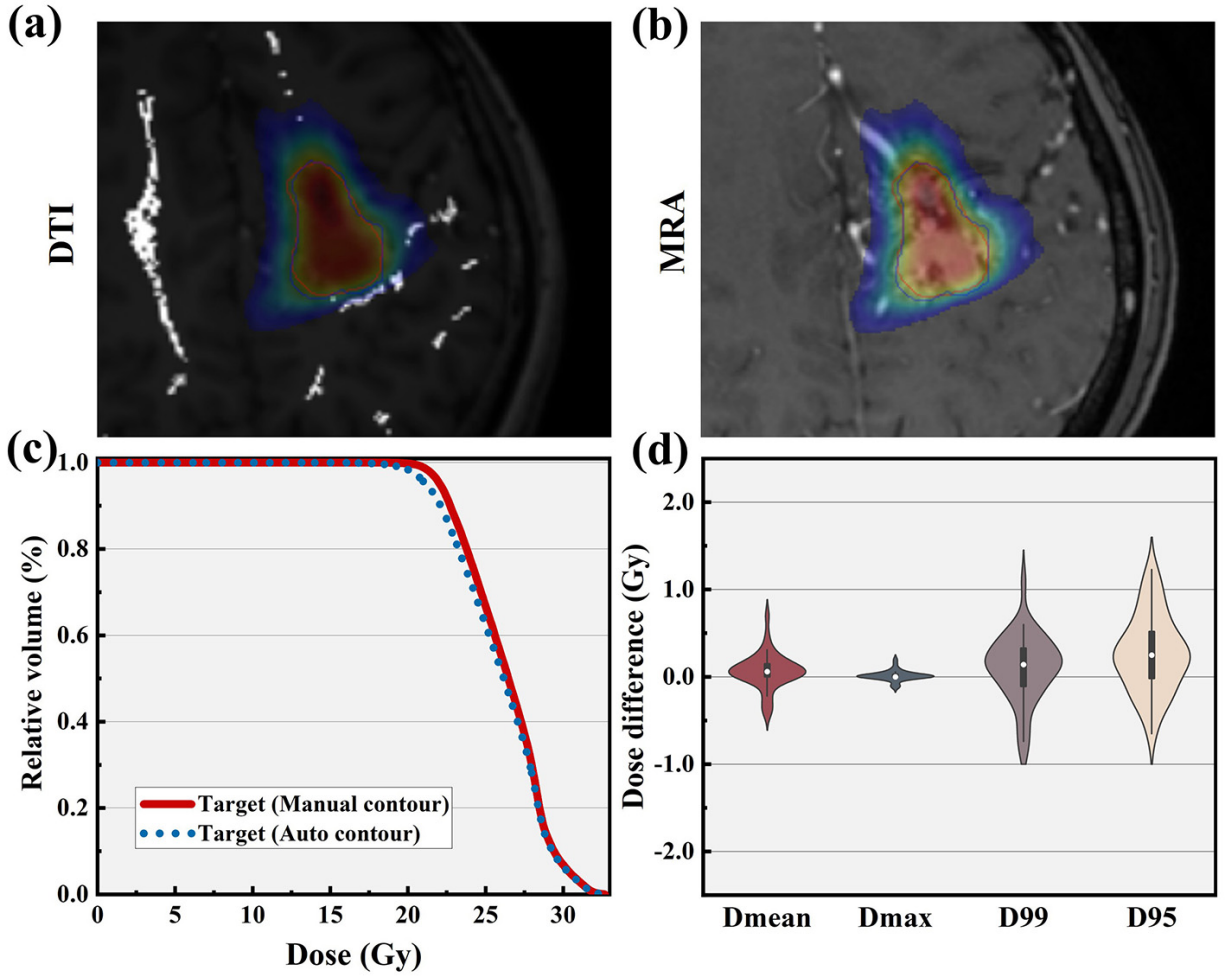
Example of a patient for dose distribution from the original plan in **(a)** DTI and **(b)** MRA views, highlighting target contours of ground truth (red) and auto-segmentation (blue). **(c)** DVH comparison for these target contours, with ground truth in red and auto-segmentation in blue. **(d)** DVH metrics comparison for the bAVM target across 38 patients in the test dataset.

An exemplary dosimetric comparison is shown in Figures 4a, b for DTI and MRA views, respectively. The corresponding DVH curves for bAVM target coverage, both manually and auto-segmented, are nearly identical, as illustrated in Figure 4c. The differences in target dosimetric parameters for 38 patients are depicted in Figure 4d. A strong concordance is observed between the auto-segmented and the manually contoured across the majority of dosimetric endpoints. Specifically, the differences in Dmean, Dmax, D99, and D95 are less than 0.46, 0.15, 1.09 Gy, and 1.23 Gy, respectively.

**Figure 5:**
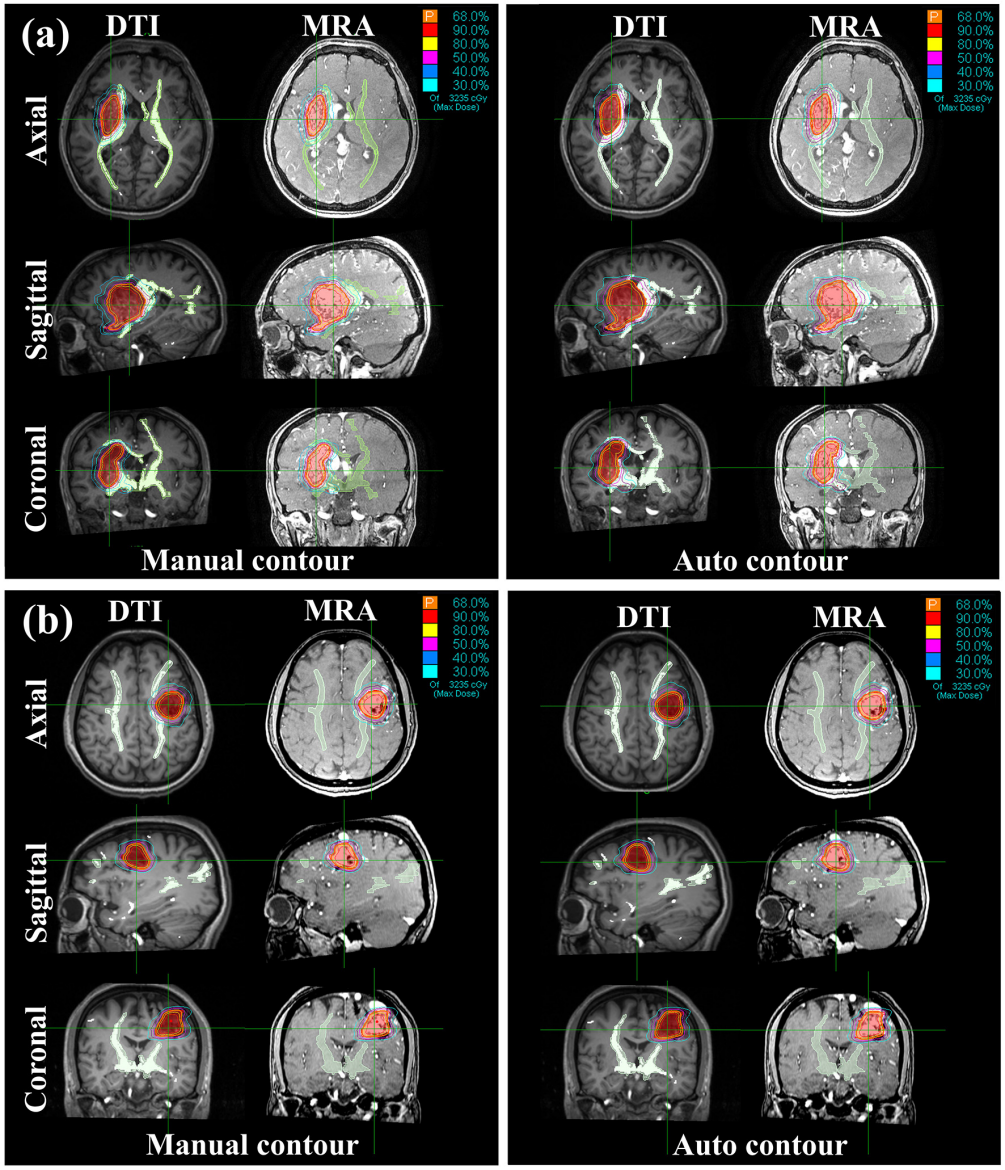
Treatment plans based on manual and auto-segmented PTV contours for **(a)** a right-sided lesion and **(b)** a left-sided lesion, incorporating DTI and MRA and displayed in axial, sagittal, and coronal planes.

**Figure 6:**
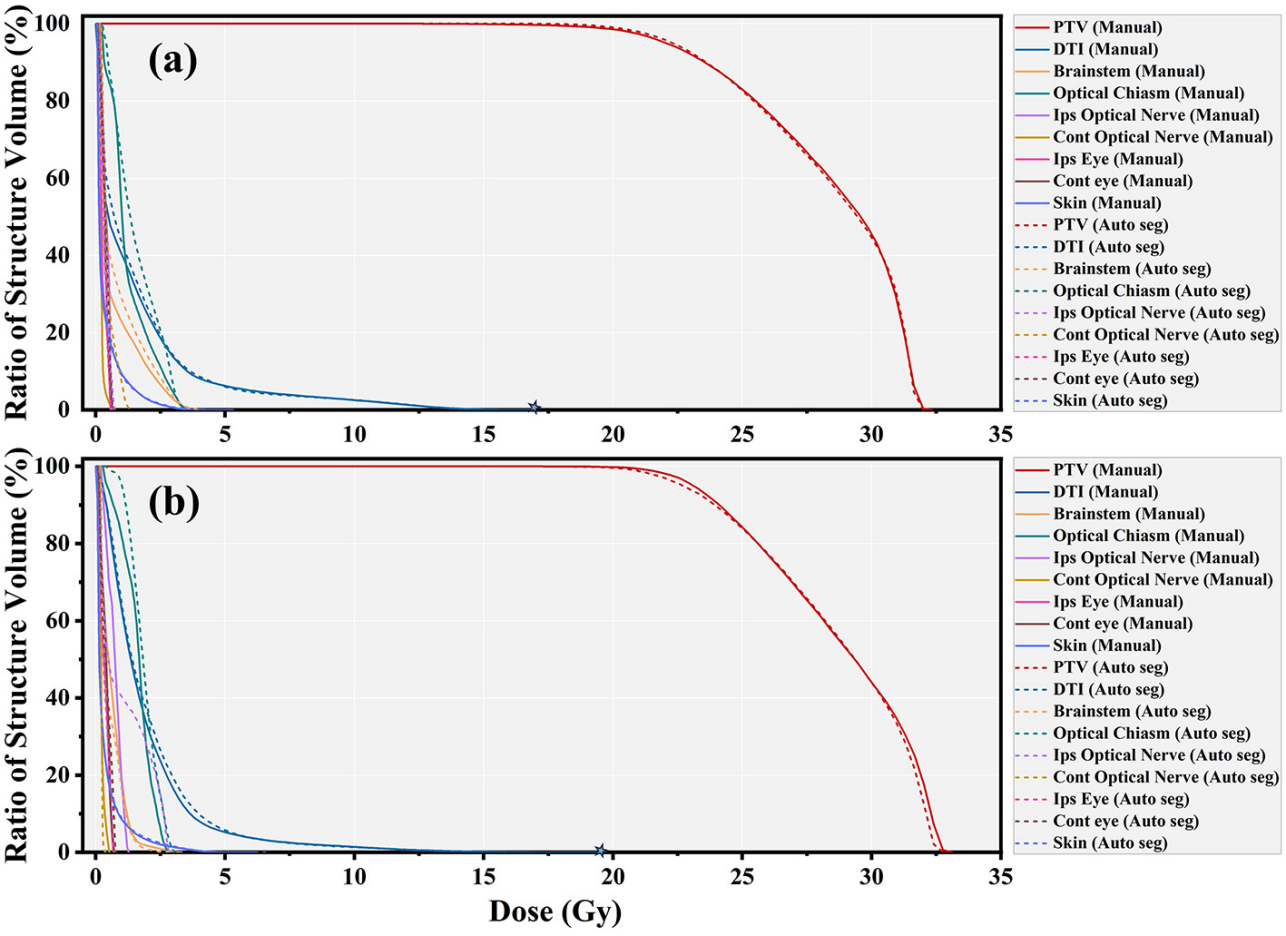
DVH obtained with manual contours (solid lines) and auto-segmented contours (dashed lines) for the PTV, corresponding to the two cases in Figure 5. **(a)** Right-sided lesion. **(b)** Left-sided lesion.

**Figure 7:**
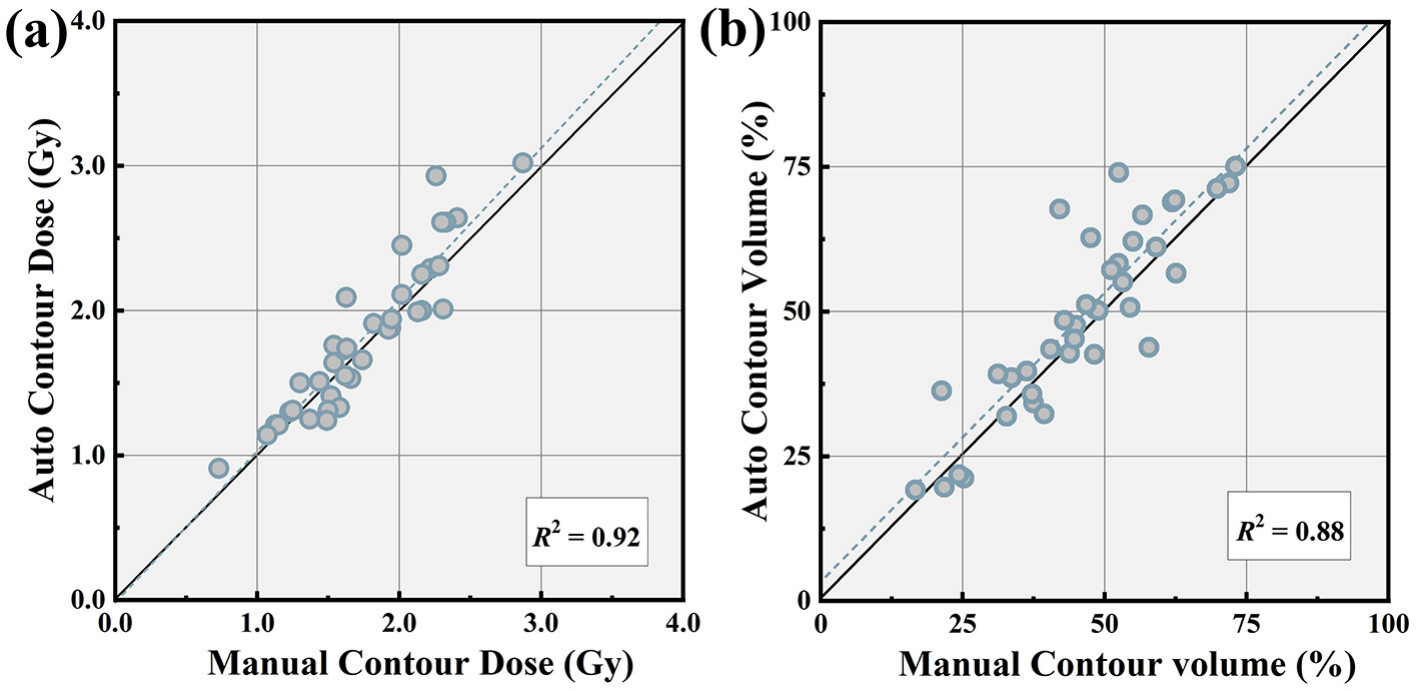
Comparison of Dmean **(a)** and V1Gy **(b)** for WM tracts in the treatment plans designed based on manually entered and auto-segmentation contoured targets for all patients in the test dataset. The dashed line represents the linear regression.

Analysis of the two treatment plan parameters revealed no statistically significant differences in target coverage (97.35 ± 3.42 vs. 97.94 ± 3.19) and the number of beams utilized (183 ± 27 vs. 175 ± 37). Similarly, the conformity index (CI), new conformity index (nCI), and Monitor units (MU) demonstrate no discernible differences between the plans. A detailed comparison is depicted in Table 5. Two representative comparison images of the two treatment plans, designed using model-generated contours and manual contours, are presented in Figure 5, plotted in axial, sagittal, and coronal planes. Corresponding DVH curves are exhibited in Figure 6. When considering the dosimetric parameters for the critical volume of WM tracts, we observe a high degree of concordance between control plans designed based on manually and auto-segmented bAVM targets. This agreement is notable for most of the WM tracts, with Dmean (*R*^2^ = 0.92) and V1Gy (*R*^2^ = 0.88), as shown in Figures 7a, b. Dose exposition of the OARs is assessed for comparison of both treatment plans. Brainstem Dmean (0.63 ± 0.41 vs. 0.67 ± 0.38 Gy, *p* = 0.02), optic chiasm Dmean (0.21 ± 0.15 vs. 0.23 ± 0.13 Gy, *p* = 0.38), and all other OARs listed in Table 6 show no statistically significant differences in treatment plans with auto-segmentation targets compared to regular plans.

**Table 5:** Plan details with manual and auto-segmentation targets.

**Parameters**	**Manual-segment**	**Auto-segment**	***p*-value**
Target coverage (%)	97.35 ± 3.42	97.94 ± 3.19	0.2
Number of beams	183 ± 27	175 ± 37	0.04
CI	1.21 ± 0.13	1.19 ± 0.09	0.17
nCI	1.28 ± 0.15	1.24 ± 0.13	0.21
MU	18,442 ± 3,581	17,936 ± 2,944	0.01

**Table 6:** Dose exhibition of organs at risk.

**OAR**	**Side**	**Dose metric**	**Manual segment (Gy)**	**Auto-segment (Gy)**	***p*-value**
Brainstem		Dmean	0.63 ± 0.41	0.67 ± 0.38	0.02
Optical chiasm		Dmean	0.21 ± 0.15	0.23 ± 0.13	0.38
Optical nerve	Ipsilateral	Dmean	0.84 ± 0.52	0.77 ± 0.69	0.11
	Contralateral	Dmean	0.24 ± 0.08	0.46 ± 0.1	0.15
Eyes	Ipsilateral	Dmean	0.32 ± 0.72	0.38 ± 0.51	0.19
	Contralateral	Dmean	0.25 ± 0.42	0.33 ± 0.27	0.2

## 4 Discussion

Accurate segmentation of bAVMs remains clinically challenging due to intrinsic complexities: heterogeneous nidus composition (encompassing abnormal vasculature, parenchyma, cerebrospinal fluid, and embolized vessels) and poorly defined lesion boundaries. To address this, we developed a cascade segmentation framework comprising a 2D U-Net for preliminary localization followed by a 3D self-attention network for precise volumetric delineation. This approach demonstrated superior segmentation accuracy for bAVMs adjacent to WM tracts, yielding a mean DSC of 0.84 ± 0.05. These results significantly outperform all existing benchmarks, including the state-of-the-art nnU-Net (0.79 ± 0.13), ResNet (0.73 ± 0.14), and standard U-Net architectures (0.76 ± 0.10). Moreover, the model's performance closely aligned with the interobserver variability across all evaluated geometric metrics. This suggested that within the intra-institutional experiments, the model has exhibited a proficiency level comparable to that of expert neurosurgeons and radiation oncologists.

While existing literature addresses bAVMs' auto-segmentation (Jiao et al., 2022; Hong et al., 2024; Wang et al., 2019; Wu et al., 2021), this study presents the first automated framework, to our knowledge, designed specifically for bAVMs intersecting WM tracts using integrated DTI and multi-modal imaging. Our methodology employs DTI co-registered with T1-weighted MRI to reconstruct WM tracts volume, enabling precise spatial mapping between critical fiber tracts and nidal boundaries. This capability directly enhances functional preservation strategies in SRS planning. We introduce the concept of WMT to evaluate the differences in WM tracts delineation between auto-segmented and manually contoured targets. As shown in Table 2, there is no statistical difference in WMT. It was clear that the target area delineated by the model effectively avoided the WM tracts, closely matching the accuracy of manual delineation. In addition, accurate bAVMs' contouring can be implemented by using multimodality images to identify features such as location, size, and invasion area (Ishihara et al., 2020). Therefore, TOF-MRA, T2-weighted MRI, and CE-CT images are utilized to provide comprehensive morphological data, including vascular structure, abnormal vascular connections, and the presence of edema and inflammation in the surrounding tissues.

The current study integrates multiple innovations to improve segmentation performance. First, departing from conventional whole-volume processing, we implement a cascaded framework: a 2D detection network generates preliminary localization of the bAVMs, identifying their spatial coordinates and boundaries. Subsequently, an optimized 3D self-attention network performs fine-grained segmentation exclusively within these regions. This targeted approach eliminates extraneous tissue interference, thereby significantly improving segmentation accuracy compared to traditional methods. Second, unlike prior U-Net-based solutions, we introduce spatial and channel attention mechanisms within a unified Attentional Block architecture. This design optimizes feature discrimination while maintaining parameter efficiency. The ablation study confirms the critical role of the attention mechanisms, as their removal led to marked performance drops across all metrics. This validates that the attention modules are essential for capturing fine structural details and achieving accurate boundary segmentation. Third, mitigating overfitting is essential given the limited clinical dataset (*N* = 191). Spatial transformations, including random deformations and rotations, are applied during training to diversify the sample population. This augmentation strategy enhances model robustness to anatomical variability while minimizing the risk of overfitting.

The inability to visualize WM tracts on conventional CT/MRI images increases the risk of critical areas adjacent to the bAVMs being subjected to excessive radiation levels, potentially inducing side effects that exceed tolerable limits. As we know, this study marks the first exploration of the effects of an automated contouring model on radiation dose estimates for both the bAVMs and WM tracts. The mean differences in DVH metrics are all less than 0.46 Gy. When considering the dosimetric parameters generated in contrasted plans using auto-segmented and manually contoured targets, we observe no significant differences in most of the dosimetric endpoints for the WM tracts. This further confirms the limitation of WM tracts dose reception for the treatment plan designed based on the target area delineated by our method, thereby achieving the protection of WM tracts in bAVM SRS.

This study has several limitations: First, the single-institution design limits demographic and technical heterogeneity. Multicenter validation is essential to confirm generalizability and enhance the robustness of the automated segmentation framework. The primary reason for this current limitation stems from the highly specialized nature of bAVM treatment using CyberKnife radiosurgery. To the best of our knowledge, this procedure is currently performed by only two institutions in China: Huashan Hospital and Tiantan Hospital. This extreme concentration of cases makes an initial single-center study a practical necessity, though we agree it introduces a constraint on generalizability. Therefore, as an immediate and concrete step to address this limitation, we are actively collaborating with Tiantan Hospital to initiate a multicenter validation study. We aim to collect an independent external dataset to further evaluate and improve the generalization capability of our model. Second, the modest dataset size may constrain model generalizability despite augmentation strategies. Third, our study is limited by the imbalanced distribution of Spetzler–Martin grades in our cohort, particularly the underrepresentation of Grade IV bAVMs. This reflects their inherent rarity but may impact the model's performance on these complex subtypes. While our initial analysis on the limited available cases showed promising results, future validation on a larger, more balanced multicenter dataset is essential to confirm generalizability across all grades. Despite these limitations, our research introduces a new neural network structure, which demonstrates promising results in both geometric metrics and dosimetric endpoints for the precise automatic segmentation of bAVMs.

## 5 Conclusion

This study presents a novel two-stage deep learning approach that integrates DTI-based WM tractography and multimodal imaging for the automatic segmentation of bAVM adjacent to WM tracts. Our work directly addresses the clinical challenge of time-consuming manual segmentation in stereotactic radiosurgery planning by developing an efficient automated solution. Validated on a clinical cohort of 191 bAVM patients treated with CyberKnife radiosurgery, the proposed method combines a detection-aided model with 3D self-attention mechanisms to achieve precise contour delineation. A comprehensive evaluation on an independent test set (*n* = 38) demonstrated high segmentation accuracy through both geometric and dosimetric analyses. Despite promising results, we acknowledge limitations such as the imbalanced distribution of Spetzler–Martin grades and the single-center study design. Future work will focus on multicenter validation with more balanced clinical cohorts and the development of real-time segmentation capabilities for clinical implementation. This approach demonstrates strong potential for supporting stereotactic radiosurgery planning for bAVMs by providing reliable dosimetric endpoints, thereby helping to avoid neurological sequelae through enhanced protection of white matter tracts while significantly improving operational efficiency.

## Data Availability

The original contributions presented in the study are included in the article/Supplementary material, further inquiries can be directed to the corresponding author/s.
