# Supplementary material for: Geometric and dosimetric evaluation of auto-segmentation of brain arteriovenous malformations using multimodal imaging in stereotactic radiosurgery
Source: Front Neurosci. 2025 Oct 30;19:1645990. doi: 10.3389/fnins.2025.1645990 (PMC12611903; doi:10.3389/fnins.2025.1645990)
Supplement: Supplementary file 1 [file Table_1.docx]

**Supplemental Table 1.** Information About Prescription Dose, Fraction, and Collimators.

| **Frontal** |  |  |  |  |  |  |  |  |  |  |  |  |  |
| --- | --- | --- | --- | --- | --- | --- | --- | --- | --- | --- | --- | --- | --- |
| Patient number | 1 | 2 | 3 | 4 | 5 | 6 | 7 | 8 | 9 | 10 | 11 | 12 | 13 |
| Prescription Dose | 21 | 21 | 21 | 21 | 21 | 22 | 22 | 21 | 22.5 | 22 | 21 | 17 | 23 |
| Fractions | 3 | 3 | 3 | 3 | 3 | 2 | 2 | 3 | 2 | 2 | 3 | 2 | 2 |
| Size of Collimators | 15 | 15, 25 | 10, 15, 25 | 7.5, 15 | 7.5, 15 | 7.5, 15 | 12.5 | 15 | 10, 15 | 12.5 | 10, 15 | 5, 12.5 | 7.5, 15 |
| **Parietal** |  |  |  |  |  |  |  |  |  |  |  |  |  |
| Patient number | 14 | 15 | 16 | 17 | 18 | 19 | 20 | 21 | 22 | 23 | 24 | 25 |  |
| Prescription Dose | 20 | 22.5 | 23 | 23 | 21 | 22 | 23 | 20 | 24 | 23 | 22 | 21 |  |
| Fractions | 4 | 3 | 2 | 3 | 3 | 3 | 2 | 4 | 2 | 3 | 2 | 3 |  |
| Size of Collimators | 12.5, 25 | 15, 25 | 10 | 7.5, 15 | 15, 25 | 10, 15 | 12.5 | 15, 20 | 5, 7.5 | 7.5, 12.5 | 7.5, 15 | 7.5, 15 |  |
| **Basal Ganglia** |  |  |  |  |  |  |  |  |  |  |  |  |  |
| Patient number | 26 | 27 | 28 | 29 | 30 | 31 | 32 | 33 | 34 | 35 | 36 | 37 | 38 |
| Prescription Dose | 21 | 22.5 | 21 | 21 | 24 | 24 | 21 | 15 | 21 | 22.5 | 21 | 16 | 22.5 |
| Fractions | 3 | 2 | 3 | 3 | 2 | 4 | 3 | 3 | 3 | 3 | 2 | 2 | 3 |
| Size of Collimators | 10, 20, 30 | 10, 15 | 7.5, 12.5 | 10, 20, 35 | 10 | 10, 20 | 10, 20, 30 | 15, 20 | 7.5, 12.5 | 7.5, 15 | 12.5, 15 | 7.5 | 10, 20 |

Prescription Dose (Gy), Size of Collimators (mm)
